# Can Consumers Make Affordable Care Affordable? The Value of Choice Architecture

**DOI:** 10.1371/journal.pone.0081521

**Published:** 2013-12-18

**Authors:** Eric J. Johnson, Ran Hassin, Tom Baker, Allison T. Bajger, Galen Treuer

**Affiliations:** 1 Center for Decision Sciences and Graduate School of Business, Columbia University, New York, New York, United States of America; 2 Center for Rationality, The Hebrew University of Jerusalem. Jerusalem, Israel; 3 Law School, University of Pennsylvania, Philadelphia, Pennsylvania, United States of America; 4 Department of Psychology, Columbia University, New York, New York, United States of America; 5 Abess Center for Ecosystem Science & Policy, University of Miami, Coral Gables, Florida, United States of America; Centre national de la recherche scientifique, France

## Abstract

Tens of millions of people are currently choosing health coverage on a state or federal health insurance exchange as part of the Patient Protection and Affordable Care Act. We examine how well people make these choices, how well they think they do, and what can be done to improve these choices. We conducted 6 experiments asking people to choose the most cost-effective policy using websites modeled on current exchanges. Our results suggest there is significant room for improvement. Without interventions, respondents perform at near chance levels and show a significant bias, overweighting out-of-pocket expenses and deductibles. Financial incentives do not improve performance, and decision-makers do not realize that they are performing poorly. However, performance can be improved quite markedly by providing calculation aids, and by choosing a “smart” default. Implementing these psychologically based principles could save purchasers of policies and taxpayers approximately 10 billion dollars every year.

## Introduction

Currently tens of millions of Americans, along with members of Congress and their staffs, are participating in a grand experiment in consumer choice: They will select health insurance using a marketplace or *health insurance exchange* operated by states and federal governments as part of the Patient Protection and Affordable Care Act. The success of these exchanges depends upon two related premises: First that consumers will be able to select the best policy for their needs, and second that price competition, driven by effective consumer choice, will lower prices. This hope is shared by divergent participants: Kathleen Sibelius, Secretary of Health and Human Services, and a Democrat, characterizes an exchange as “… a transparent, level playing field, driving down costs; … giv[ing] individuals and small businesses the same purchasing power as big businesses and a choice of plans to fit their needs.” [1] Bill Frist, a physician and former Republican Senate Majority Leader, argues “State exchanges are good from a conservative standpoint because they involve consumer choice and markets.” [2].

These premises are critical not only to the new exchanges, but also for all government administered health insurance markets and for the efficiency of privately provided benefit choices – both health exchanges and employer sponsored insurance center on consumer choice in finding plans that are cost-effective and appropriate for consumer needs and both include many design decisions that will affect choice. Yet a large literature in psychology suggests that these premises may not be realistic, since, as we shall see, these exchanges may not provide a helpful *choice architecture* to support decision-making. In this paper, we examine three related questions: Can people select the best policies? Do they know how well they are doing? Does the design of the sites change their performance?

Our results suggest there is significant room to improve these decisions. Without any intervention, respondents perform at near chance levels and show a significant bias, overweighting out-of-pocket costs and deductibles. Financial incentives do not improve performance, and decision-makers do not realize that they are performing badly. Without aids, only one population examined here, Columbia MBA students, perform reasonably well at this task. However, performance *can* be markedly improved by providing calculation aids, and by choosing a “smart” default, raising the performance of ordinary respondents to that of the MBA students.

### Prior Research

The quality of choices on prior health insurance exchanges has been, at best, mixed. For example, when examining the exchanges implementing Medicare Part D, a prescription drug plan for seniors, Heiss, McFadden, and Winter [3] conclude, “consumers are likely to have difficulty choosing among plans to fine tune their prescription drug coverage.” Abaluck and Gruber [4] find that only 12.2% of seniors pick the most cost-effective plan.

While the economic analysis of choice suggests that issues surrounding incentives and information may determine success, a more psychological analysis suggests that good decisions depend, critically, on subtle elements of how the choices are presented to the consumer, as described in an evolving literature on choice architecture [5]–[7]. Designing an exchange involves many design decisions including specifying the number and kind of options and attributes offered, determining the arrangement of options and the format and order of attributes, and selecting default options and computational aids.

The Massachusetts “Connector,” an exchange operating since 2006, illustrates the impact of choice architecture: Before late 2009, the Connector simultaneously presented 25 plans from 6 insurance providers. In 2009, plans were reorganized into 3 tiers of coverage, categorized by premiums and out-of-pocket costs. Consumers first chose one of these levels and then viewed a smaller set of 6 standardized plans within a level. Work by Ericson and Starc [8] shows that this simple change markedly altered behavior: Consumers were increasingly sensitive to premium costs and out-of-pocket costs, changing market shares for some carriers by a factor of 2.

Thus, the advent of health exchanges presents a challenge: The choice could be daunting for consumers, resulting in suboptimal choices of policies that provide the wrong features or are too expensive. We are interested in how a prudent design of health exchanges based on psychological research could improve choice. We are also interested in a parallel question: Do people know if they are making good decisions? This is important because if people know that they are not doing well, they could seek assistance, potentially remedying their poor performance. If people are unaware of their inadequate performance, simply providing access to assistance will not improve their decision-making.

## Methods: Choosing health insurance

When choosing insurance, consumers face two tasks. The first, which we do not examine, is to estimate their expected usage and out-of-pocket expenses for the upcoming year, and to consider the uncertainty around these quantities as a choice under risk. The second is to select the right plan given their expected usage.

Our studies focused on people's ability to select cost-effective policies and remove risk and usage prediction considerations. While economists analyze insurance choice by examining uncertainty, risk, and asymmetric information, we investigated the impact of psychological variables such as calculation costs as a major barrier to better choices. We examined a simplified version of the health insurance choice that allowed us to assess the performance of choice architecture interventions, much like a wind tunnel might be used to evaluate candidate airplane designs – see Methods S1 for a thorough explanation of the experimental procedure.

Even in this simplified version of health insurance choice, the process of selecting the most cost-effective health insurance is not trivial, consumers must, for each plan:

1. Consider the total premiums for the year,
2. Combine the copayments and the expected number of visits, and
3. Include the minimum of the deductible and their out-of-pocket costs.

For equal monthly premiums this is (12*Monthly Premium) + (N of visits * Copay) + min(Out-of-Pocket Costs, Deductible). Adding risk considerations, while undoubtedly important, would make these calculations even more difficult, thus making performance worse, not better than we observe, and perhaps make our interventions more effective.

**Figure 1:**
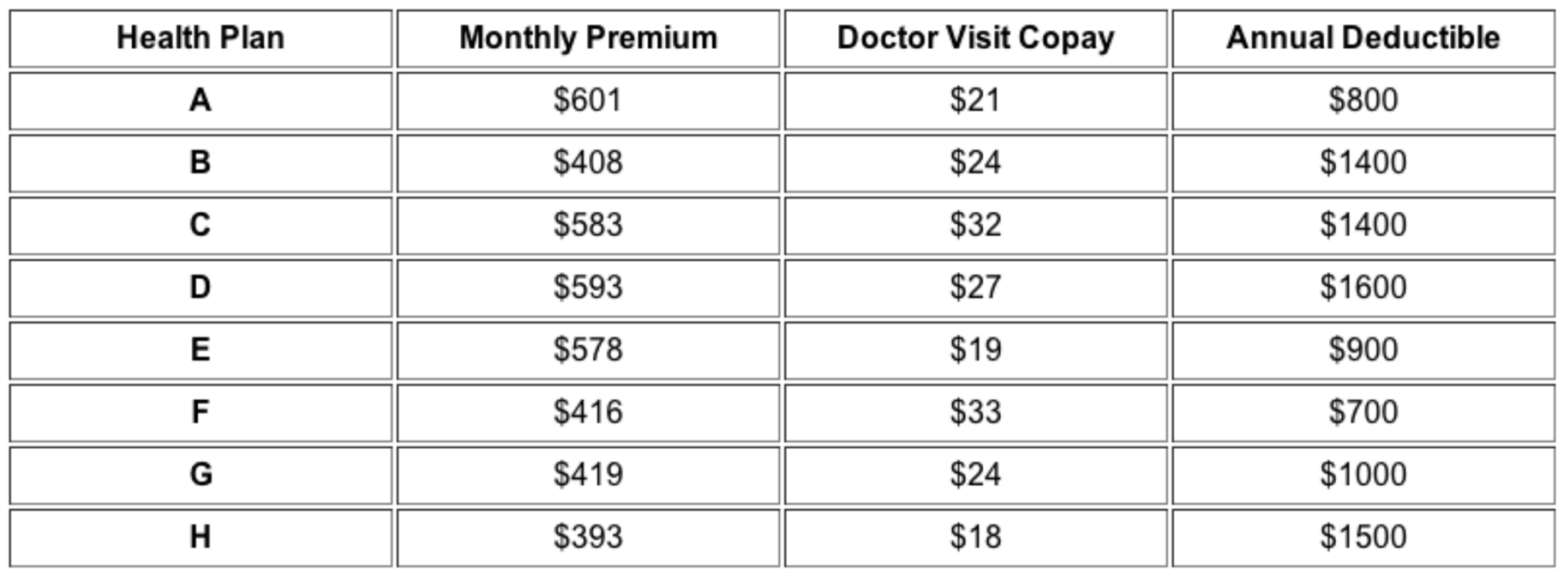
A decision display used in Experiment 4. Respondents saw either 8 (pictured) or 4 options.

The reader might consider selecting the most cost effective plan in Figure 1, assuming, as did respondents in one of our experimental conditions, that he or she will make 9 doctor visits and incur $900 in out-of-pocket costs in the upcoming year. This calculation might seem difficult, but some would argue that there might be heuristic strategies that perform well [9]. Yet we feel that there are two reasons for concern: First, users of these exchanges will be largely unfamiliar with selecting health insurance – since many, 97% according to some estimates [7], will be buying health insurance for the first time and may lack experience and relevant knowledge – and will not be highly educated (seventy-seven percent will have a High School diploma or less) [10]. Second, this is an economically significant decision for these households: Even with subsidies, premiums will represent between 4 and 9.5% of the modest median income of $48,529 for a family of 4 [10]. Consequently, mistakes may have large economic consequences.

## Results and Discussion: Can Consumers Choose The Right Plan?

We examined consumers' decision-making abilities and conditions that might facilitate better decisions in a series of six framed field experiments [11], all but one using participants with demographics similar to those projected to use the exchanges. In addition to specifying the number of doctor visits one would make and the out-of-pocket costs one would incur in a given year, we also limited the number of plans available to either 4 or 8, a figure markedly lower than the number to be used in future exchanges (e.g., the Massachusetts Connector currently presents 47 plans, a discussion of choice set size) [12].

In all six experiments, subjects were asked to imagine they were choosing health insurance for a family of three – themselves, a partner, and one child – with an anticipated number of doctor visits and out-of-pocket health care costs over the next year. Each subject was required to choose one plan from a set of 4 plans and one from a separate set of 8 plans. Plan set order was counter-balanced so half of the subjects chose from the 4-plan set first and half chose from the 8-plan set first. Within each set of 4 and 8 plans, the display order of plans was also varied. In some experiments the number of visits or anticipated costs were varied (described below).

All studies shared certain features: All responses were collected online (see Table S1 for demographics and other details). To isolate the effect of making a choice from a misunderstanding of the basic mechanics of health insurance, each session included explanations about insurance terms, such as premium, co-pay, and deductible, and required respondents to pass a comprehension test before proceeding (see methodological details in Methods S1 for the content of these instructions and tests). Only those participants who passed this test were included in our analyses. Respondents viewed a table modeled after prototypes of exchanges (Figure 1) and chose an insurance plan. In Experiment 1 and 2, all components of prices resembled current prices and relationships among prices seen in existing and prototype exchanges. In addition, Experiments 1–2 varied, between respondents, the number of visits, while Experiments 3–5 varied the level of out-of-pocket costs. For the sake of brevity, we will not discuss these results here.

**Figure 2:**
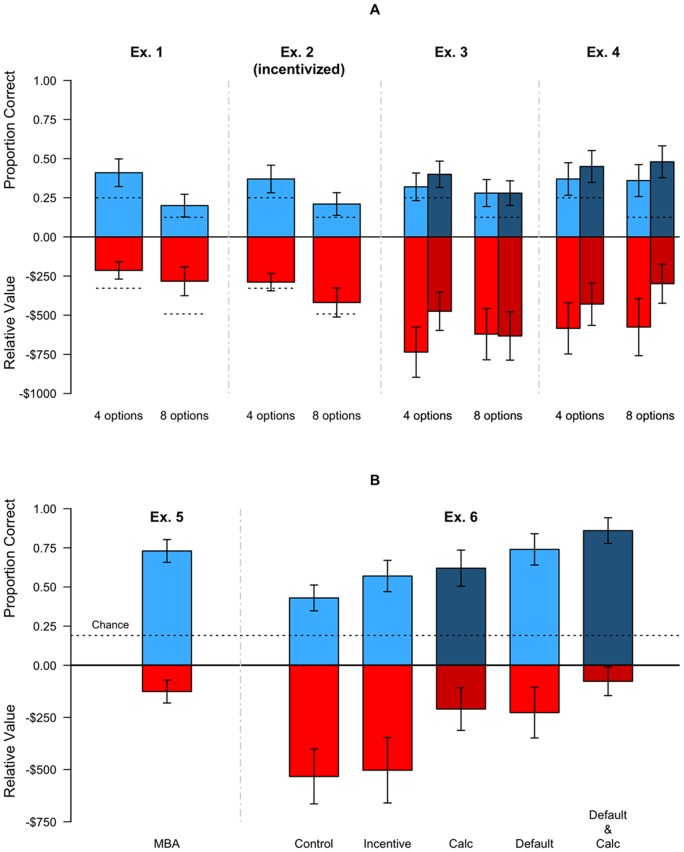
The percentage of choices of the most cost effective option and respondents' average error. The top half of each bar, in blue, represents the proportion of correct choices, and the bottom half, below the zero line in red, plots the average dollar error, across respondents. A dashed line for each condition represents the performance of a random chooser, and the error bars represent 95% confidence intervals. Darker shades denote the provision of calculators. Panel (A) represents the results of Experiments 1–4 collapsing across other manipulations (see SM). Panel (B) represents the results of a sample of highly educated MBA students (Experiment 5), and of individuals from the target population, when given different choice architecture interventions. For (b) the random response threshold ($1264) exceeds the lower limit of the graph.

Experiment 1 provided a baseline measure of the proportion of people who choose the most cost-effective policy from 4 or 8 options. Figure 2 shows the outcomes from all experiments. The top half of each bar, in blue, represents the proportion of correct choices, and the bottom half, in red, plots the average dollar error, across respondents. We model all choices using a logistic model with indicator variables for categorical variables, and an Analysis of Variance to test significance for the error cost variable – please see Methods S1 for more details. The dashed line represents expected choice quality by a random chooser. Panel A of Figure 2 shows a rather dramatic outcome: With 4 choice options, respondents selected the best option only 42 percent of the time, and made an average mistake of over $200 dollars. With eight options, they selected the correct option 21 percent of the time, a figure not different than chance (*p*>.05).

Experiment 2 added monetary incentives: Selecting the most cost-effective policy increased payment by $1 and generated an entry to a lottery that paid $200 to one correct chooser – including the lottery, the expected value of selecting each right option was $1.88, and performance was unrelated to time spent on the task. As can be seen in the next two bars of Figure 2 (A), incentives did not improve outcomes, and performance was close to chance.

This failure might be due to individuals' inability to perform the daunting calculations. One obvious intervention, used in Experiments 3 and 4, involves the use of a cost-calculator stating the annual total cost. In fact, several existing web sites, including Medicare.gov, provide such a tool. The present studies emphasized another important change designed to help diagnose the cause of poor performance: Plan attributes were drawn from an orthogonal experimental design, allowing us to estimate the weight participants give to the three cost components, premiums, co-payments and deductibles. According to economic theory, these costs should be approximately equally weighted since they all occur over the course of a year, and all contribute to the annual cost of the policy. However, past research has indicated that some costs (usually deductibles) are overweighted while others, like premiums are underweighted [4], [13], [14]. In addition, Experiment 4 also simplified the choice by removing quality information for half of the respondents – this information was not diagnostic, since all options had the same total quality, and the choices made by respondents confirmed this.

The results, shown in the third and fourth columns of Figure 2 (A), are not markedly different. Again respondents chose the most cost effective option less than half the time, and made large financial errors. The unaided decision-makers averaged errors of $611 in Experiment 3 and chose the correct option 32% of the time. Providing the calculators marginally helped but only in Experiment 4: Respondents provided with calculators chose the correct option 10.1% more often, and reduced the size of errors by $216, but still were only correct 47% of the time and made mean errors of $364.

**Figure 3:**
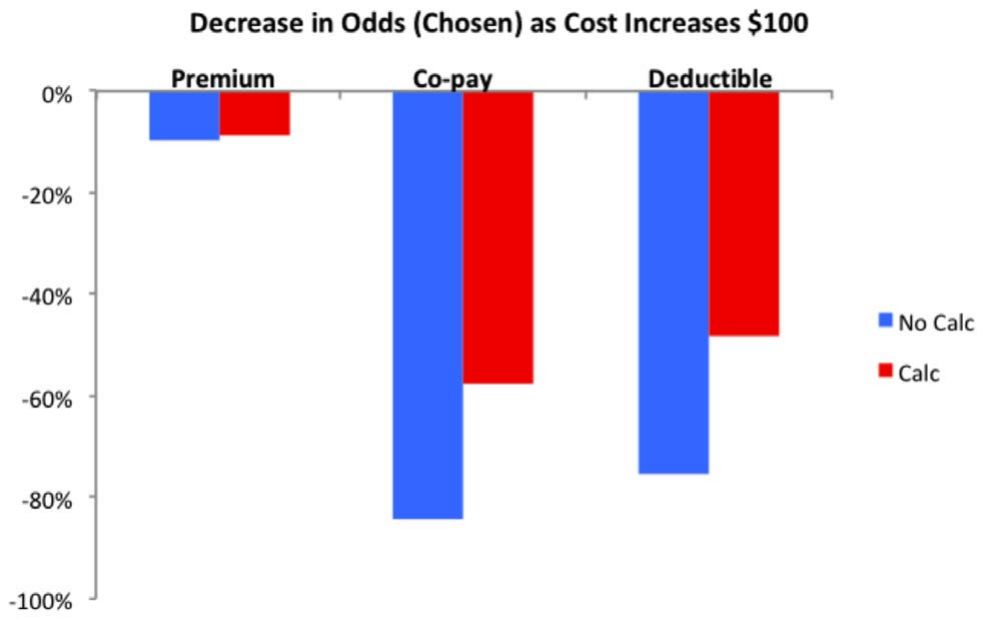
Premiums, deductibles, and co-payments, both without calculator (blue) and with calculator (red). The decline in odds of being chosen for each increase in $100 in annual cost for the three cost components in experiments.

Why was performance so poor? Answering this question may suggest interventions. While the math alone is challenging, the failure of the calculator to improve choice suggests that something else may be going on. Recall that past research shows that deductibles may be overweighted [13]–[16]. If this is the case, consumers may, arguably, have an incorrect notion of how deductibles contribute to overall cost. Figure 3 shows the weight given to each price component in Experiment 4. The results show a strong and consistent bias, compared to the ideal of equal weighting: Participants overweight the out-of-pocket costs and deductibles. Their improved performance with calculators is due, in part, to reducing this bias, as illustrated by the red bar. In other words, the presence of a calculator suggests that respondents came closer to treating all dollars as having the same cost.

Is this task simply impossible? Experiment 5 used a very different population to see how highly trained, financially literate individuals might do. We presented MBA students enrolled in a class on consumer finance with the same task as in Experiment 4. The average GMAT of students at this school was 716, and 59% of students came from consulting or financial services and related fields. As seen in the first column of Figure 2(B), they performed appreciably better, choosing the right option 73% of the time, and making an average mistake of $126. Their self-reports of how they accomplished the task are interesting: Forty percent reported using excel (this group performed quite well, selecting the correct option 85% of the time, and making an average error of only $47). This suggests that having *both* the right mental model *and* the ability to execute these calculations may be a basic requirement to make good choices.

In Experiment 6, we explored the possibility that mental models in conjunction with different possible interventions would produce good performance by individuals who will be using the exchange. To ensure understanding, and encourage the use of the correct mental model, all conditions received a tutorial about computing the annual cost and completed a quiz requiring one correct choice. We believe that this kind of *just-in-time* education might help both aided and unaided choice, and further eliminate a lack of knowledge (as opposed to computational complexity) as a barrier to better performance. We then compared this control condition to four different manipulations. An incentive group received a more extreme and sophisticated incentive regime that penalized respondents 10 cents for every $100 extra that was spent on insurance. We contrasted this to three choice architecture interventions. The first provided a *calculator*, explained what the calculator did, and tested that understanding. The second provided a *smart default* that preselected the most cost effective options given individuals' usage. Individuals could, and did, change that selection if desired. Finally, we combined *defaults and calculators*. The presence of incentives and our choice architecture manipulations allowed us to compare the cost effectiveness of these interventions.

The last four bars in Figure 2(B), which average data over the number of options, show that the treatments vary widely in effectiveness. The controls, despite having received instruction and tests of understanding, chose about as well as respondents in prior experiments. The second bar indicates that incentives did not have a significant effect on outcomes, even though individuals in the incentive condition took 38% longer to make their decisions, a significant increase relative to controls. Calculators (with education), in contrast, produced better decisions, having resulted in a significant decrease in the size of the loss and an increase in the proportion correct. The smart default option had a similar effect, as it reduced losses and increased the percentage correct. It is important to note that the performance of defaults is not simply due to their mindless selection. First note that a significant proportion of people (21%) chose to not take the default by actively selecting another option. Second, those choosing the default option did take a significant amount of time to choose a policy. Across the entire study, non-default choosers required 443 seconds to complete the study, and choosers required 348 seconds. Concentrating on only the choice screen, default choices took 58% and 65% as long as the no-default condition for the 4 and 8 option conditions, respectively. Finally, when combined, the defaults and calculators seemed to complement each other, leading to performance levels that are comparable to those of the highly trained MBA students. This last result suggests, perhaps, that because calculators provide a justification for the default, they increase the transparency of their selection, and increases their adoption. It also suggests that providing just-in-time education along with calculation and choice aids produces better performance.

While these interventions are effective, are they appreciated? This is an important question about meta-cognition that has important policy implications: If deciders are doing badly and need help, do they realize it? When they get help, do they appreciate it? We asked respondents how confident they were of making the correct choice in Experiments 3, 4 and 6, using a 1–9 point scale: While participants performed poorly, this was not reflected in their confidence ratings (mean rating 6.6, 6.75, and 7.6, respectively, in Experiment 3, 4, and the control condition in Experiment 6) and there was no correlation between these ratings and selecting the most cost effective plan (.09 averaged across these three studies). It appears that individuals did not realize the need for these interventions. They also did not appreciate the effect of the interventions consistently: Calculators created a marginal increase in confidence (+.23 relative to control, *p*<.06); defaults did not (+.14, *p*>.2). Finally, incentives did not increase performance, but they did increase effort and produced an unwarranted increase in confidence (+.34, *p*<.03). All told, the picture that emerges is that of overconfident decision-makers who do poorly and do not realize it, and who do not realize that decision-architecture helped.

## Conclusions

Our results present a bad news/good news story of particular importance. The bad news: Consumers left to their own devices seem to make large errors when choosing health insurance, suggesting that they will select options that are not cost-efficient and they seem to be unaware of their failure. If consumers cannot identify cost-efficient plans, then the exchanges will not produce competitive pressures on health plan costs, one of the main advantages of relying upon choice and markets. It is possible that other factors, such as advertising and brokers may make the market more or less competitive. The impact of such institutions is a question for further research.

The good news is that we have demonstrated that exchange designers can improve consumers' performance markedly through the use of just-in-time education, smart defaults, and cost calculators. This list of potential design improvements is not exhaustive, and there are many other interventions that may improve choices. These include sorting by cost, the presence of quality cues, or limiting the number of options to those that meet criteria of cost-effectiveness. These suggestions are not without precedent: In evaluations of Medicare Part D, Abaluck and Gruber [4] suggest that “restricting the choice set to the 3 lowest average cost options would have likely raised welfare for the elders.” However, this limits consumer choice and we note that some design features, such as calculators, improve outcomes by making choice easier, without impinging upon consumer sovereignty.

The results of these studies allow us to approximately estimate the benefits of these kinds of choice architecture interventions. These estimates should be treated with appropriate caution because they are based on the particular set of policies used in our studies. However, our control group in Experiment 6 made an average error of $533, roughly 10% of the cost of the cheapest policy, compared to an error of $77 when both the default option and calculator were available, producing an estimated value to these features of $456 dollars per decision. At the individual level, unaided choice is expensive: It represents about 1% of the income of the proposed median buyers' household income. But in the aggregate, an error of $456 represents staggering sums: If 20 million individuals make choices using the exchanges, a figure suggested by Congressional Budget Office estimates, unaided choice represents a cost to consumers of $9.12 billion dollars each year. Since almost all of these policies are subsidized through tax credits, good choice architecture would produce substantial savings to the federal budget and taxpayers.

This sizable impact is more significant since the improvement is largely a function of psychological factors that can be implemented inexpensively by being built into the choice engines powering the exchanges. Clearly, further research identifying the best mix of choice architecture tools in exchanges is both scientifically interesting and economically justified. While the success of the health exchanges will depend, in part, on the provision of cost-efficient products, it also will depend on the design of exchanges that will allow consumers to identify the best choice that is a good fit to their needs. Ignoring the impact of choice architecture and the psychological factors we examine could be an expensive mistake.

## Supporting Information

Table S1 **Demographics.**
(DOCX)

Methods S1 **Methods and Materials.**
(DOCX)
