# Supplementary material for: Can Consumers Make Affordable Care Affordable? The Value of Choice Architecture
Source: PLoS One. 2013 Dec 18;8(12):e81521. doi: 10.1371/journal.pone.0081521 (PMC3867314; doi:10.1371/journal.pone.0081521)
Supplement: Table S1 — Demographics. (DOCX) [file pone.0081521.s001.docx]

Table S1**.**

*Demographics*

| ­ | **Experiment 1** | **Experiment 2** | **Experiment 3** | **Experiment 4** | **Experiment 5** | **Experiment 6** |
| --- | --- | --- | --- | --- | --- | --- |
| N | 120 | 131 | 234 | 177 | 76 | 330 |
| Age (mean) | 48 | 48 | 45 | 37 | 29 | 50 |
| Gender | Female (72%) | Female (62%) | Female (56%) | Female (55%) | Female (46%) | Female (64%) |
| Marital Status | Married (56%) | Married (47%) | Married (47%) | Married (40%) | Married (32%) | Married (44%) |
| Children | 0 – 2 (76%) | 0 – 2 (75%) | 0 – 2 (79%) | 0 – 2 (87%) | 0 – 2 (4%) | 0 – 2 (74%) |
| Income | $35K - $49K (51%) | $50K - $99K (26%) | $50K - $99K (27%) | $50K - $99K (28%) | > $99K (35%) | $20K - $49K (67%) |
| Education | HS diploma (35%) | HS diploma (37%) | HS diploma (42%) | Bachelor’s (35%) | Master’s (66%) | HS diploma (40%) |
| Race | White (87%) | White (83%) | White (83%) | White (81%) | White (59%) | White (87%) |
| Health Insurance | Yes (66%) | Yes (66%) | Yes (75%) | Yes (65%) | Yes (97%) | Yes (72%) |
| Political Affiliation | Democrat (32%) | Democrat (30%) | Democrat (35%) | Democrat (36%) | Democrat (46%) | Democrat  (30%) |
