# Supplementary material for: Can Consumers Make Affordable Care Affordable? The Value of Choice Architecture
Source: PLoS One. 2013 Dec 18;8(12):e81521. doi: 10.1371/journal.pone.0081521 (PMC3867314; doi:10.1371/journal.pone.0081521)
Supplement: Methods S1 — Methods and Materials. (DOCX) [file pone.0081521.s002.docx]

**Supporting Information**

**Methods and Materials**

**Online surveys**

This study was approved by and carried out under Institutional Review Board at Columbia University, and all participants in each experiment provided written informed consent. Subjects were recruited from three different populations. A total of 815 subjects were recruited for Experiments 1, 2, 3 and 6 by third party survey companies from a demographic population similar to the one likely to use online insurance exchanges. 177 subjects were recruited for Experiment 4 through Amazon Mechanical Turk (MTurk), restricted to Americans in the 97^th^ percentile of all workers. 76 MBA students from a major research university were recruited for Experiment 5 through a class exercise. In each experiment subjects completed an online survey that included three sections: 1) an introduction to health insurance policies and comprehension quiz; 2) the choice task in which subjects were instructed to imagine they were buying health insurance for their family of three with specific expected health costs and choose one of several plans; and 3) post-task individual difference questions covering strategies used, demographics and personal health insurance information.

**Incentives**

Two different incentive schemes were employed. In Experiment 2, subjects received an additional $1 for answering correctly on each of the choice questions. In addition, participants were given an entry into a $200 drawing for each correct answer. If participants chose the most cost-effective option for both choice questions, they received $2 and two entries into the $200 drawing. In Experiment 6 we attempted to increase the incentive through loss aversion and tying payment to the cost of the plans subjects chose. A portion of the subjects were endowed with $7 which was reduced by 10 cents for each $100 difference in annual cost between the insurance plan they chose and the cheapest plan.

**Preliminary Data Analysis**

To ensure attention, each subject was required to pass a version of Oppenheimer and colleagues’ Instructional Manipulation Check [1] before learning about health insurance policies. Subjects were removed from analysis for two reasons: 1) if they failed to demonstrate understanding by correctly answering multiple choice questions about health insurance policies in three tries, and 2) if they completed the decision task too quickly (below the 5^th^ percentile or 10 seconds) or too slowly (above the 99^th^ percentile or above 1100 seconds). 57 subjects (32%) were excluded from Experiment 1 and 74 (36%) from Experiment 2 for failing to demonstrate understanding. 166 subjects (41%) were excluded from Experiment 3, 34 for time, 55 for failing to demonstrate understanding and 11 for both. 18 (10%) from Experiment 4 were excluded, 10 for time, 2 for failing to demonstrate understanding and 6 for both. No subjects were excluded from Experiment 5 and 117 (26%) from Experiment 6, 17 for time, 79 for failing to demonstrate understanding and 21 for both. The variation in exclusion rates is consistent with anticipated differences in online experimental populations. Analyzing the data with the excluded respondents does not result in meaningful changes, but does add increased random error in almost all of our measures.

**Experimental Designs**

In all six experiments, subjects were asked to imagine they were choosing health insurance for a family of three, themselves plus a partner and one child, with an anticipated number of doctor visits and out-of-pocket health care costs over the next year. Each subject chose one plan from a set of 4 plans and one from a separate set of 8 plans. Order was counter-balanced so half of the subjects chose from the 4 plan set first and half chose from the 8-plan set first. Within each set of 4 and 8 plans the order plans were displayed was also varied. In some experiments the number of visits or anticipated costs were varied, described below.

Experiments 1 and 2 were designed to reflect the design of real insurance policies with negatively correlated premiums and deductibles. In Experiments 3, 4, 5 and 6 an orthogonal experimental design was used, in which all cost components of the plans presented are uncorrelated. This enabled us to estimate the weight given to the three cost components: premium, co-payment, and deductible. Quality information was provided for respondents in Experiments 1-3 and half of respondents in Experiment 4. This information was designed to be independent of the price components. To simplify the choice, quality was not presented in subsequent experiments. Experiment 4 allowed us to establish that the quality measures had no effect on performance.

**Data analysis**

Data was analyzed in the *r* software package [2] using analysis of variance (Type III, marginal sum of squares), with the size of the error made as the dependent variable, or the equivalent model as a binomial logistic regression, with selection of the cheapest plan (yes or no) as the dependent variable. All models included as factors choice set (4 options or 8 options) and order presented (first or second). Copay usage (high or low) was included as an additional factor in Experiments 1 and 2, and out of pocket expense (high or low) was included in Experiments 3, 4, and 5. Annual cost calculator (present or not) was included in Experiments 3, 4, 5, and 6. In an alternative model incentive and default plus annual cost calculator were included as contrasts to the control group (no calculator, no default, no incentive) in Experiment 6.

**Attribute Analysis**

We also sought to understand the importance of the cost measures associated with each plan in driving selection of a given plan, and especially how the importance of these measures varied depending on the values of the experimental variables in a given choice scenario. In order to address these questions, we fit a logistic regression on a transformed version of the original dataset in which each choice among a choice set of k options was represented with k-1 rows and the dependent variable was defined as whether or not a given choice had been selected in that choice set. The most expensive option was the reference category; due to multicollinearity among the choice measures in the 4-option grid, analyses were restricted to the 8-opton grid data.

**Detailed Results**

**Experiment 1**

Subjects were assigned to high (15) or low (5) number of doctor visits. Overall 30% (chance = 19%) chose the most cost-effective option and made an average mistake of $248 (chance = $410). Given 4 options 41% (chance = 25%) chose the most cost-effective option and made an average mistake of $214 (chance = $328). Given 8 options 20% (chance = 12.5%) chose the most cost-effective option and made an average mistake of $283 (chance = $492). Analysis of variance (ANOVA) with the size of error made as the dependent variable found a main effect of usage (*F*(1,229) = 11.77, *p* < 0.001) and no significant effect of grid (*F*(1,229) = 1.68) or order presented (*F*(1,229) = 0.08). High usage (15 visits), with 4 options 69% chose the most cost-effective plan and made an average mistake of $89, with 8 options 38% chose the most cost-effective plan and made an average mistake of $196. Low usage (5 visits), with 4 options 19% chose the most cost-effective plan and made an average mistake of $311, with 8 options 6% chose the most cost-effective plan and made an average mistake of $351.

**Experiment 2**

Experiment 2 used the same design as Experiment 1 with the added $1 per correct answer and $200 lottery incentive described above, in the methods section. Overall 29% chose the most cost-effective option and made an average mistake of $354. Given 4 options 37% chose the most cost-effective option and made an average mistake of $289. Given 8 options 21% chose the most cost-effective option and made an average mistake of $419. ANOVA with size of error made as the dependent variable found a main effect of usage (*F*(1,249) = 12.75, *p* < 0.001), a marginally significant effect of grid (*F*(1,249) = 3.58, *p* < 0.1) and no effect of order presented (*F*(1,249) = 0.37). High usage (15 visits), with 4 options 57% chose the most cost-effective plan and made an average mistake of $150, with 8 options 34% chose the most cost-effective plan and made an average mistake of $323. Low usage (5 visits), with 4 options 17% chose the most cost-effective plan and made an average mistake of $427, with 8 options 8% correct and made an average mistake of $515.

Combining Experiment 1 and 2, a logistic regression with choice of the cheapest option as dependent variable found main effects of grid *X*^2^ (1,*N* = 502) = 23, *p* < 0.001 and usage *X*^2^ (1,*N* = 502) = 90, *p* < 0.001 but not order *X*^2^ (1,*N* = 502) = 0.1 or incentive *X*^2^ (1,*N* = 502) = 1.3, results further supported by binomial order regression. An ANOVA with size of error made as dependent variable found a significant (negative) main effect of the incentive added in Experiment 2 (*F*(1,481) = 7.75, *p* < 0.01) as well as usage (*F*(1,481) = 24.4, *p* < 0.001) and grid (*F*(1,481) = 5.27, *p* < 0.05) but not order presented (*F*(1,481) = 0.1). Incentives added in Experiment 2 are associated with a $118 *increase* in losses. Though they did not help them perform better, incentives did significantly increase the time subjects spent choosing a plan from a mean of 85 second to 98 second (*t*(230) = -2.1, *p* < 0.05).

**Experiment 3**

In Experiment 3 doctor visits were fixed at 11, and subjects were assigned to high ($2200) or low ($900) anticipated out-of-pocket expenses. Half of subjects had the total annual cost of each plan calculated and presented along side the three cost components. Overall performance was poor with 32% choosing the most cost-effective option and an average mistake of $611 (chance = $1262). Given 4 options 36% chose the most cost-effective option and made an average mistake of $594 (chance = $1191). Given 8 options 28% chose the most cost-effective option and made an average mistake of $627 (chance = $1337). In the high expense condition 29% chose the most cost-effective option and made an average mistake of $562. In the low expense condition 35% chose the most cost-effective option and made an average mistake of $654. Adding an annual calculator helped non-significantly. With a calculator 34%, compared with 30% without, chose the most cost-effective option, and the average mistake was reduced by $123. A logistic regression with an ANOVA test for significance on choice of the cheapest option found a main effect of grid *X*^2^ (1,*N* = 468) = 4, p < 0.05 and no significant effect of order *X*^2^ (1) = 0.06, expense *X*^2^ (1) = 2.3 or annual calculator *X*^2^ (1) = 1.4. An ANOVA on the size of error made found no main effects of grid (*F*(1,426) = 0.2), order (*F*(1,426) = 1.2), expense (*F*(1,426) = 0.8), or annual calculator(*F*(1,426) = 1.8).

**Experiment 4**

In Experiment 4 the design of Experiment 3 was implemented with a more highly educated and online-experienced MTurk population. For half of the subjects, non-informative quality ratings were removed to reduce noise, which had a no significant effect on performance. Though the MTurks did better than previous subjects, overall only 42% chose the most cost-effective option and the average mistake was $466. Given 4 and 8 option grids subjects performed virtually the same. 41% and 42% chose the most cost-effective option respectively and made average mistakes of $502 and $429. Subjects provided with annual cost calculations performed significantly better, 47% vs. 37% without the calculations and an average reduction in mistakes made of $216. Subjects in the high expense ($2200) condition, in which the deductible level was reached with every plan, performed significantly better than those in the low expense ($900) condition. 48% vs. 36% chose the most cost-effective option with a $186 average reduction in mistake made. These differences in means are supported by a logistic regression with an overall ANOVA test on choice of the cheapest option that found a main effect of annual calculator *X*^2^ (1) = 3.86, *p* = 0.05 and expense *X*^2^ (1, *p* < = 0.05) but no significant effect of grid *X*^2^ (1) = 0.2, quality ratings *X*^2^ (1) = 2.7, p = 0.1 or order *X*^2^ (1) = 1.3. Binomial order regressions provide further support for these findings. Similar results are found in MANOVA with mistake made. Annual calculator (*F*(1,325) = 7.8, *p* < 0.01) and expense (*F*(1,325) = 6.32, *p* < 0.05) are significant but grid (*F*(1,325) = 1.27), quality ratings (*F*(1,325) = 1.75), and order (*F*(1,325) = 1.69) are not.

The attribute importance analysis in Study 4 revealed that participants weighted a $100-increase in annual copay cost and annual deductible cost significantly more than a similar increase in annual premium cost (Figure 3). A $100 increase in premium cost was associated with a 6% decrease in the odds of selecting a given plan (OR= .94, 95% CI: .89-.99); a $100 increase in copay cost with a 57% decrease in the odds of choosing a plan (OR= .43, 95% CI: .12-1.58); and a $100 increase in deductible cost with a 61% decrease in odds (OR= .39, 95% CI: .16-.87). Interaction effects suggested that subjects weighted premium more in the calculator condition (OR= 1.89, 95% CI: 1.80-1.99, with calculator vs. OR= .94, 95% CI: .90-.99, without calculator, premium*calculator interaction *p*= .06). Copay was weighted less in the second trial (*p*= .01) and deductible was weighted less in the low expense condition (*p*= .002).

**Experiment 5**

To investigate if anyone can successfully perform this task, in Experiment 5 MBA students completed Experiment 4 with two changes. Subjects were instructed to “choose the most cost-effective plan” and quality ratings were removed in all conditions. The MBA students performed much better than other populations. Overall 73% chose the most cost-effective option with an average loss of $126. Given 4 and 8 options subjects performed virtually the same. 72% and 75% chose the most cost-effective option respectively with averages mistakes of $157 and $95. The addition of an annual cost calculator had no significant effect. This may be because 86% of the MBA students reported using Excel, a calculator or pen and paper to find the cheapest option. Subjects in the high expense ($2200) condition, in which the deductible level was reached with every plan, performed significantly better than those in the low expense ($900) condition. 83% vs. 64% chose the most cost-effective option with a non-significant $65 average reduction in mistakes made. Though identifying the cheapest option appears to be easier in the high expense condition, with the use of excel and other tools the MBA students were still able to minimize their losses in the low expense condition.

A logistic regression with choice of the cheapest option and an ANOVA on the size of error made as dependent variables support these results.

**Experiment 6**

In an attempt to get subjects who fit the profile of people likely to use the health insurance exchanges to perform as well as MBA students, we administered a version of Experiment 5 to an online panel with incomes under $50,000. To increase power, all subjects were assigned to the high expense ($2200) condition. An incentive-compatible condition (described above) was administered to 47% of the participants. 46% were provided with annual cost calculations.

In the control condition (no calculator, no default, no incentive) 43% chose the most cost-effective option, similar to overall performance in Experiment 4, with an average mistake of $533. In the incentive compatible condition (no calculator, no default) 57% chose the most cost-effective option but average mistake dropped by a non-significant $30 (*t*(203) = 0.28, *p* = 0.8). Providing annual cost calculations increased choice of most cost-effective option to 62% and decreased the average mistake by $323. Default selection of the most cost-effective option significantly increased its selection to 74% and decreased the average mistake by $306. The combination of annual cost calculator and defaults significantly increased selection of the most cost-effective option to 86% and significantly decreased the average mistake by $456.

Logistic regression on choice of the cheapest option supports these findings. Default *X*^2^ (1) = 39, p < 0.001 and annual calculator *X*^2^ (1) = 8.9, p < 0.01 are significant while grid *X*^2^ (1) = 0.0, incentive *X*^2^ (1,N = 660) = 0.9 and order *X*^2^ (1) = 0.18 are not, results also found in binomial order regressions. ANOVA on size of error made also support significant positive impacts of the default (*F*(1,640) = 17, *p* < 0.001) and annual calculator (*F*(1,640) = 27, *p* < 0.001). Grid (*F*(1,640) = 4.1, *p* < 0.05) was also significant, but incentive (F(1,640) = 0.02) and order (*F*(1,640) = 0.05) were not.

The individual effect of defaults, annual calculators, and incentives were further confirmed through contrasts analysis in regression controlling for grid and order. Incentives improve choice of the cheapest option *X*^2^ (1,N = 660) = 9.9, *p* < 0.01 but not mistake made (*F*(1,642) = 1.5). Smart defaults improved both choice of the cheapest option *X*^2^ (1) = 29, *p* < 0.001 and size of error (*F*(1,642) = 18, *p* < 0.001). The annual calculator also improved both choice of the cheapest option *X*^2^ (1) = 17, *p* < 0.001 and size of error made (*F*(1,642) = 19.6, p < 0.001). Defaults combined with annual calculator improved helped subjects perform even better in choosing the cheapest option *X*^2^ (1) = 51, *p* < 0.001 and reducing mistakes (*F*(1,642) = 38.5, *p* < 0.001).

References

1. Oppenheimer DM, Meyvis T, Davidenko N (2009) Instructional manipulation checks: Detecting satisficing to increase statistical power. Journal of Experimental Social Psychology 45: 867-872.
2. R Development Core Team. (2009) *R: A language and environment for statistical computing*. Vienna, Austria: R Foundation for Statistical Computing Reference.
